# Knowledge, attitudes, and practices and long-term immune response after rVSVΔG-ZEBOV-GP Ebola vaccination in healthcare workers in high-risk districts in Uganda

**DOI:** 10.1016/j.vaccine.2024.05.079

**Published:** 2024-06-15

**Authors:** Michelle A. Waltenburg, Markus H. Kainulainen, Amy Whitesell, Luke Nyakarahuka, Jimmy Baluku, Jackson Kyondo, Sam Twongyeirwe, Jessica Harmon, Sophia Mulei, Alex Tumusiime, Eric Bergeron, Dana L. Haberling, John D. Klena, Christina Spiropoulou, Joel M. Montgomery, Julius J. Lutwama, Issa Makumbi, Alfred Driwale, Allan Muruta, Stephen Balinandi, Trevor Shoemaker, Caitlin M. Cossaboom

**Affiliations:** a Division of High-Consequence Pathogens and Pathology, Centers for Disease Control and Prevention, Atlanta, GA, United States; b Uganda Virus Research Institute, Entebbe, Uganda; c Department of Biosecurity, Ecosystems, and Veterinary Public Health, Makerere University, Kampala, Uganda; d Uganda Ministry of Health, Kampala, Uganda

**Keywords:** Ebola, Ebola vaccine, rVSVΔG-ZEBOV-GP

## Abstract

**Background:**

The rVSVΔG-ZEBOV-GP Ebola vaccine (rVSV-ZEBOV) has been used in response to Ebola disease outbreaks caused by Ebola virus (EBOV). Understanding Ebola knowledge, attitudes, and practices (KAP) and the long-term immune response following rVSV-ZEBOV are critical to inform recommendations on future use.

**Methods:**

We administered surveys and collected blood samples from healthcare workers (HCWs) from seven Ugandan healthcare facilities. Questionnaires collected information on demographic characteristics and KAP related to Ebola and vaccination. IgG ELISA, virus neutralization, and interferon gamma ELISpot measured immunological responses against EBOV glycoprotein (GP).

**Results:**

Overall, 37 % (210/565) of HCWs reported receiving any Ebola vaccination. Knowledge that rVSV-ZEBOV only protects against EBOV was low among vaccinated (32 %; 62/192) and unvaccinated (7 %; 14/200) HCWs. Most vaccinated (91 %; 192/210) and unvaccinated (92 %; 326/355) HCWs wanted to receive a booster or initial dose of rVSV-ZEBOV, respectively. Median time from rVSV-ZEBOV vaccination to sample collection was 37.7 months (IQR: 30.5, 38.3). IgG antibodies against EBOV GP were detected in 95 % (61/64) of HCWs with vaccination cards and in 84 % (162/194) of HCWs who reported receiving a vaccination. Geometric mean titer among seropositive vaccinees was 0.066 IU/mL (95 % CI: 0.058–0.076).

**Conclusion:**

As Uganda has experienced outbreaks of Sudan virus and Bundibugyo virus, for which rVSV-ZEBOV does not protect against, our findings underscore the importance of continued education and risk communication to HCWs on Ebola and other viral hemorrhagic fevers. IgG antibodies against EBOV GP were detected in most vaccinated HCWs in Uganda 2–4 years after vaccination; however, the duration and correlates of protection warrant further investigation.

## 1. Introduction

Ebola disease is a rare and often fatal illness caused by infection with an orthoebolavirus, four of which are pathogenic to humans: Ebola virus (EBOV), Sudan virus (SUDV), Bundibugyo virus (BDBV), and Taï Forest virus [1]. Ebola disease was first recognized in 1976 and outbreaks in humans have mainly occurred in Sub-Saharan Africa [2,3]. Ebola disease outbreaks have been reported in Uganda since 2000 and have been associated with BDBV (2007) and SUDV (2000, 2011, two in 2012, and 2022) [3,4]. Additionally, in June–August 2019, four cases of EBOV were confirmed in Kasese, Uganda, among individuals traveling from the Democratic Republic of Congo (DRC) [5].

The rVSVΔG-ZEBOV-GP Ebola vaccine (rVSV-ZEBOV) is a live recombinant vesicular stomatitis virus vaccine expressing the surface glycoprotein of EBOV (species *Orthoebolavirus zairense*) [6]. The vaccine is administered as a single dose and has been found to be safe and efficacious in preventing Ebola disease caused by EBOV, which has caused the largest and most deadly outbreaks to date, including the 2014–2016 outbreak in West Africa [6,7]. The vaccine is not protective against other orthoebolaviruses [8]. Since August 2018, rVSV-ZEBOV has been used in response to multiple Ebola disease outbreaks in the DRC and neighboring countries, including Uganda [5,9]. Between 2018 and 2019, 4,420 healthcare and frontline workers from 165 high-risk health facilities in Uganda near the DRC border were vaccinated under a compassionate use protocol for Ebola disease preparedness by the Ministry of Health of Uganda and the World Health Organization [10,11]. Additionally, in response to the four EBOV cases confirmed in Uganda that were attributed to cross-border movement from DRC, ring vaccination with the rVSVΔG-ZEBOV vaccine was introduced in Uganda under expanded access. Between June 15–June 26, 2019, 821 contacts and contacts of contacts, including 23 healthcare workers (HCWs), were vaccinated as part of outbreak response efforts [5].

Investigation into immune responses following vaccination with rVSVΔG-ZEBOV is critical to inform potential recommendations for booster doses. Ugandan HCWs who work in health facilities that border DRC are considered a high-risk population due to the proximity to Eastern DRC, the epicenter of recent Ebola disease outbreaks, and the continued threat of re-emergence of Ebola disease in Uganda [3,12]. We assessed knowledge, attitudes, and practices (KAP) related to Ebola disease and vaccination and evaluated the long-term immune response following vaccination with rVSV-ZEBOV among HCWs in select high-risk districts in Uganda.

## 2. Methods

### 2.1. Study population & recruitment

**Fig. 1.**
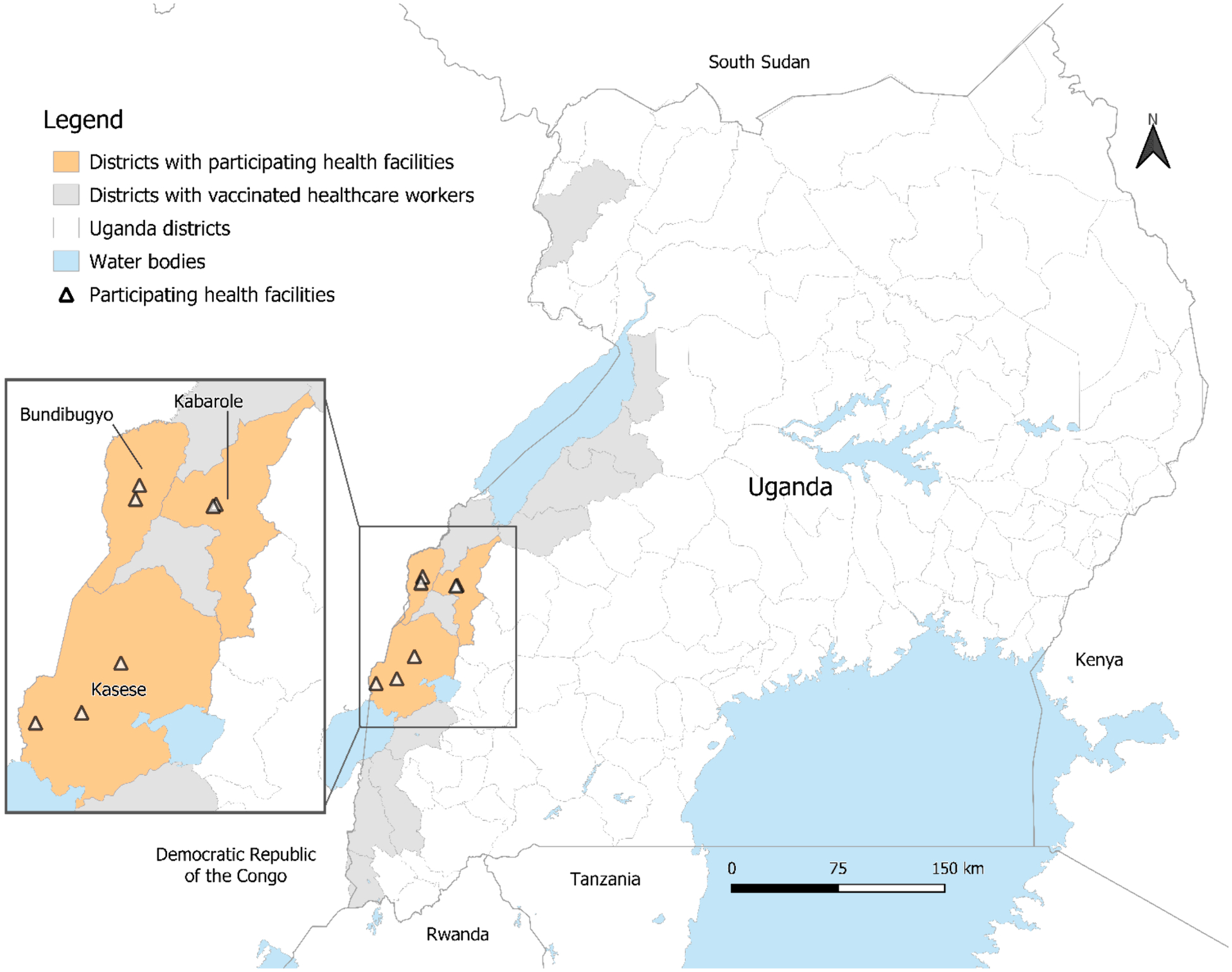
Map of participating healthcare facilities across three high-risk districts in Uganda near the Democratic Republic of Congo border.

Our study population included a convenience sample of vaccinated and unvaccinated HCWs in seven healthcare facilities across three high-risk districts in Uganda near the DRC border working in health facilities with personnel who were vaccinated with rVSV-ZEBOV in 2018–2019 under compassionate use/expanded access (Fig. 1). Study investigators, with assistance from local HCWs, used contact information for health facilities in high-risk districts to contact staff working in their facilities to inform them of the study and ask if they were interested in enrolling.

### 2.2. Ethics

Interested individuals were asked to provide written consent to enroll. All participation was voluntary; participants received a small monetary compensation for their time to participate. The Uganda Virus Research Institute Research and Ethics committee granted approval of this study on 07 October 2021 (approval number: GC/127/856). This activity was reviewed by the US Centers for Disease Control and Prevention and conducted in accordance with applicable federal law and CDC policy [13].

### 2.3. Questionnaire

The questionnaire was written in English and verbally administered by study investigators using Epi-Info^™^ 7 mobile application on tablets. We collected information on demographic characteristics, vaccination status, healthcare-associated risk factors for EBOV seropositivity, and general KAP related to Ebola and vaccination (Supplementary Data).

### 2.4. Blood collection

Participants had approximately 10 ml of whole blood collected using a serum separator tube (BD Vacutainer). Specimens were allowed to clot for one hour at room temperature (RT), then centrifuged at 2500 rpm for 10 min at RT. Aliquots of cleared serum were stored on dry ice or in freezers at −70 to −80 °C. Peripheral blood mononuclear cells (PBMC) were collected from a subset of participants in four Cell Preparation Tubes (BD Vacutainer) per participant (approximately 32 mL of whole blood). Tubes were centrifuged at 2800 rpm for 25 min at RT. PBMC layers were harvested in 2 % heat-inactivated fetal bovine serum (HI-FCS; Gibco) + 2 mM ethylenediaminetetraacetic acid (Sigma-Aldrich) in phosphate-buffered saline without calcium and magnesium (PBS, wash buffer). PBMCs were pelleted at 1200 rpm for 10 min at RT, the pellet was re-suspended in ACK red blood cell lysis buffer (Gibco) for five minutes at RT, diluted 1:10 in wash buffer, and centrifuged at 1700 rpm for five minutes at RT. The pellet was re-suspended in HI-FCS and 20 % dimethyl sulfoxide (DMSO) in HI-FCS, to obtain a final 10 % DMSO concentration. When necessary, the cells were passed through a 70 μm cell strainer to remove clumps. PBMCs were then aliquoted into 1 mL vials and frozen at a controlled rate of 1 °C/min using dry ice when −80 °C freezers were unavailable. Thereafter, the cells were stored at vapor phase nitrogen, −70 °C freezers, or dry ice until permanent storage facility with vapor phase nitrogen could be reached.

### 2.5. Enzyme-Linked immunosorbent assay to detect IgG antibodies against EBOV glycoprotein

ELISA was performed at CDC essentially as previously described [14]. Briefly, stabilized EBOV strain Kikwit glycoprotein (GP) trimer was produced in Expi293 cells and C-terminally tagged with biotin. MaxiSorp 384-well plates (Nunc) were coated with 25 ng StrepTactin (IBA Lifesciences) in PBS over night at + 4 °C. The plates were washed three times with 0.1 % Tween-20 (Sigma-Aldrich) in PBS (PBS-T) to remove excess StrepTactin and blocked with 5 % (w/V) milk powder (Cell Signaling Technology) in PBS-T at RT. After discarding the blocking solution, 12.5 ng C-terminally biotinylated antigen was captured in the blocking buffer for antigen capture by StrepTactin-biotin interaction. Blocking solution without antigen was added to no-antigen control wells. After washing to remove excess antigen, a 7-point, 3-fold dilution series of heated serum samples (+56 °C, 30 min) was prepared in blocking buffer and added in duplicate (final sample dilutions 1:100 to 1:72,900). The 1:100 dilution of each sample was also added to no-antigen wells. In each plate, a dilution series of an in-house standard was also analyzed. After 1 h incubation at RT and 3 PBS-T washes, secondary goat anti-human IgG antibody conjugated to horseradish peroxidase (Jackson ImmunoResearch) diluted 1:10,000 in blocking buffer was added for 1 h at RT. The further 3 washes with PBS-T, 1-Step Ultra TMB substrate (Thermo Fisher) was added for 10 min, and reactions were stopped by addition of ELISA Stop Solution (Invitrogen) before quantifying absorbances at 450 nm. Optical density values (OD) were corrected by subtracting average signals from no-sample wells, and then against individual sample background by using the ratio of 1:100 signal from antigen-containing wells and no-antigen control wells.

After a preliminary analysis of a subset of the samples, a corrected OD value of 0.35 was chosen as positivity cut-off (3.6 standard deviations above average of 54 unvaccinated participant samples with specificity of 98.1 %, and 100 % sensitivity among 11 vaccinated participants that presented a vaccination card). To further assess the specificity of the assay, serum samples from U.S. donors (n = 92), known to be negative for Ebola virus antibodies, were tested and all were found to test negative, yielding specificity of 100 % in that sample set. For samples determined to be positive, the dilution with OD closest to value 1 (which falls within the linear range of the OD measurements) was compared to the linear portion of the plate-specific standard in-house standard curve (log-transformed values). These values, representing fold-over in-house standard, were transformed into International Units (IU)/mL by using fold-difference of the in-house standard and the WHO International Reference Reagent WHO15/220 (The National Institute for Biological Standards and Control, UK). This fold difference was derived as the average from 3 independent runs. Comparisons to the FANG ELISA assay were done using the reported FANG titer for the same standard [15].

### 2.6. Neutralization assay

Virus neutralization assays were performed as previously described [14]. In brief, serum samples were heated to +56 °C for 30 min and dilutions prepared in medium containing 2 % heat-inactivated FCS. Ebola virus strain Mayinga expressing the green fluorescent protein [16] was then mixed with diluted serum samples so that 40 μL of this mix contained approximately 150 focus-forming units (FFU) of infectivity. The mixes were incubated for 1 h at +37 °C and triplicate 96-wells of Vero-E6 cells infected with 40 μL after removing the growth medium. After 1 h at +37 °C with periodical shaking, the inocula were removed and the cells overlayed with medium containing 1 % (w/V) carboxymethyl cellulose (Sigma-Aldrich) and 4 % HI-FCS. Fluorescent foci were counted after a 5-day incubation at +37 °C, 5 % CO2 using a Cytation 3 instrument and Gen5 software (BioTek).

The samples were initially screened for neutralization at 1:10 and 1:20 final serum dilutions. Having noticed that inhibition close to 50 % can be present in some negative samples, only samples that inhibited virus infectivity by > 80 % were considered positive for neutralization. Those samples initially screened as inhibiting virus infectivity by > 70 % were repeat tested using a 7-point dilution series from 1:10 to 1:640. For positive samples, the titer that inhibited focus formation by 50 % (focus-reduction neutralization titer, FRNT_50_) was determined from log-transformed concentrations using a four-parameter nonlinear curve fit of the GraphPad Prism software package.

### 2.7. Elispot assay

PBMCs were thawed rapidly at +37 °C, washed twice with 10 % HI-FCS + 2 mM GlutaMax + 100 U/mL penicillin + 100 ug/mL streptomycin in RPMI-1640 medium (Gibco) and incubated in the same over-night at +37 °C, 5 % CO_2_. Cell number and viability were then determined using Guava ViaCount assay and easyCyte 8HT instrument (Luminex). To be included in assays, samples had to reach > 80 % viability and quantity of > 2.5 million cells / mL. Samples that failed to meet these criteria were repeat thawed using another aliquot. Human IFN-γ ELISPot assay (MabTech) was run according to manufacturer’s recommendations. Triplicate wells of 250,000 live PBMCs were stimulated with a 74-peptide mixture comprised of strongly stimulating pools GP1-1, GP1-2, GP1-3 and GP2-2 (minus peptides EB-GP-Z-45, −50, −51 and 184–187 due to manufacture and solubility issues) [17], each peptide at a final concentration of 7.5 μg/mL. To determine background activity, cells were stimulated with DMSO vehicle (final concentration 0.6 %). As a positive control for stimulation, 25,000 cells were stimulated with the kit’s CD3 mAb positive control in duplicate. The plates were stained 18 h after beginning stimulation according to the manufacturer’s instructions and imaged after drying using an ImmunoSpot S6 Entry analyzer (CTL). Average spot counts resulting from peptide stimulation were corrected by subtracting DMSO spot counts and expressed as spot-forming cells (SFC) / 1 million cells. Cut-off value of 5,000 SFC/million for CD3 stimulation was applied to exclude non-responsive samples. Repeat testing of samples not reaching the quantity/viability cut-off was attempted and the data represents averages from 1 to 3 independent experiments/sample.

### 2.8. Statistical analyses

Basic descriptive statistics for categorical variables were calculated as frequencies and continuous variables were expressed as median and interquartile range (IQR). We assessed differences in frequency distribution between groups using Chi-square tests and Fischer’s exact tests when the expected cell size was < 5.

As vaccination history was self-reported, we categorized participants into four groups: 1) provided a vaccination card; 2) did not provide a vaccination card but provided the name and estimated date of vaccine; 3) did not provide vaccination card but provided the name or estimated date of vaccine; 4) did not provide a vaccination card, name, or estimated date of vaccine.

All statistical analyses of the KAP section were carried out using R statistical software (version 4.2.2; R foundation) and maps were generated using ArcGIS version 9.3 (ESRI, Redlands, CA). Statistical analysis of the laboratory data was performed using GraphPad Prism version 9.

## 3. Results

### 3.1. Study location

Overall, 565 HCWs were enrolled from seven healthcare facilities across three districts in Uganda near the DRC border (Fig. 1). Three healthcare facilities were in Kasese District, two in Kabarole District, and two in Bundibugyo District. Most HCWs were enrolled from healthcare facilities in Kasese (55 %, 310/565), followed by Kabarole (32 %, 180/565), and Bundibugyo (13 %, 75/565) (Table 1).

### 3.2. Demographics

The median age of HCWs was 31 years (IQR: 24–43) and 53 % (300/565) were male (Table 1). Nearly all HCWs were Ugandan (>99 %, 564/565). The most reported education levels were certificate (33 %, 188/565), diploma, Bachelors, Master, or Doctoral (23 %, 130/565), and finished primary (19 %, 106/565). The most reported occupations were cleaner, plumber or catering (24 %, 136/565), nurse or midwife (23 %, 128/565), and medical student (21 %, 117/565). Most HCWs had worked in healthcare for 1–5 years (45 %, 257/565).

### 3.3. Vaccination

Among the 565 HCWs enrolled, 210 (37 %) reported any previous Ebola vaccination. Among 248 HCWs previously offered the Ebola vaccine, 38 (15 %) declined the vaccine. Overall, 193 vaccinated HCWs provided information on the type of Ebola vaccine received; of these, nearly all HCWs reported receiving the rVSV-ZEBOV vaccine (96 %, 186/193). Seven (4 %) HCWs reported receiving an Ebola vaccine other than rVSV-ZEBOV.

One third of vaccinated HCWs (33 %, 69/210) provided a vaccination card. Approximately half (54 %, 114/210) did not provide a vaccination card but provided the name and estimated date of vaccine, 11 % (24/210) did not provide vaccination card but provided the name or estimated date of vaccine, and 1 % (3/210) did not provide a vaccination card, name, or estimated date of vaccine.

### 3.4. Ebola KAP

Overall, 79 % (448/565) of HCWs could not name at least one virus that causes Ebola disease (Table 2). Compared to vaccinated HCWs (67 %), a higher percentage of unvaccinated HCWs (87 %) could not name at least one virus that causes Ebola disease (p-value < 0.01). Among the HCWs that could name at least one virus, most named EBOV (88 %, 103/117) and BDBV (78 %, 91/117).

Nearly 60 % of unvaccinated HCWs knew there was a vaccine to protect against Ebola disease prior to the survey (56 %, 200/355). Among all HCWs who were aware of the vaccine, knowledge that rVSV-ZEBOV only protects against EBOV was low among vaccinated (32 %; 62/192) and unvaccinated (7 %; 14/200) HCWs.

Most vaccinated (91 %, 192/210) and unvaccinated (92 %, 326/355) HCWs indicated that they would want to receive a booster or initial dose of rVSV-ZEBOV, respectively (Table 2). Among these HCWs, most vaccinated (74 %, 142/192) and unvaccinated (63 %, 205/326) indicated that they would want to receive a booster or initial dose of rVSV-ZEBOV immediately (Supplementary Table S1). Safety concerns (e.g., vaccine-related adverse events) were the most reported reason for hesitancy in receiving a dose of Ebola vaccine (vaccinated HCWs: 61 %, 11/18; unvaccinated HCWs: 59 %, 17/29) (Supplementary Table S2).

All vaccinated HCWs, regardless of vaccine categorization (i.e., did or did not provide a vaccination card), had higher frequencies of correct responses to KAP questions compared with unvaccinated HCWs (Supplementary Table S3). Notably, higher EBOV GP seropositivity was observed among HCWs who provided a vaccination card (Supplementary Table S3). While KAP related to Ebola and vaccination was low among HCWs overall, seropositive HCWs had higher frequencies of correct responses to KAP questions than seronegative HCWs (Supplementary Table S4).

Among 565 HCWs interviewed, 15 (3 %) reported an unprotected exposure to blood or body fluid(s) from a symptomatic, confirmed, or suspect Ebola disease patient. Of these, five HCWs reported exposure to confirmed Ebola disease patients, two HCWs reported exposure to suspected Ebola disease patients that later tested negative, and eight HCWs did not know or remember the reported exposure patient’s final diagnosis (Table 2). Eight (53 %) of these 15 HCWs reported previous Ebola vaccination. Overall, <1% (2/565) HCWs reported a prior confirmed Ebola disease diagnosis. Both survivors were exposed and diagnosed in 2007 during the outbreak in Bundibugyo, Uganda (caused by BDBV). Both later received the rVSV-ZEBOV Ebola vaccine.

### 3.5. Immune responses

Overall, 98 % (555/565) of HCWs had blood specimens collected, including 203 (37 %) vaccinated and 352 (63 %) unvaccinated HCWs. Seven vaccinated HCWs reported receiving an Ebola vaccine other than rVSV-ZEBOV and two reported surviving BDBV infection in 2007 prior to receiving the vaccine. These nine individuals were excluded from the analysis of rVSV-ZEBOV immune responses. Seventeen HCWs who reported receiving an Ebola vaccine but could not provide information on the type of vaccine received were assumed to be vaccinated with rVSV-ZEBOV for the purposes of the serologic analysis. The median time from vaccination to sample collection was 37.7 months (IQR: 30.5, 38.3) among 144 HCWs with information on month and year of vaccine receipt.

**Fig. 2.**
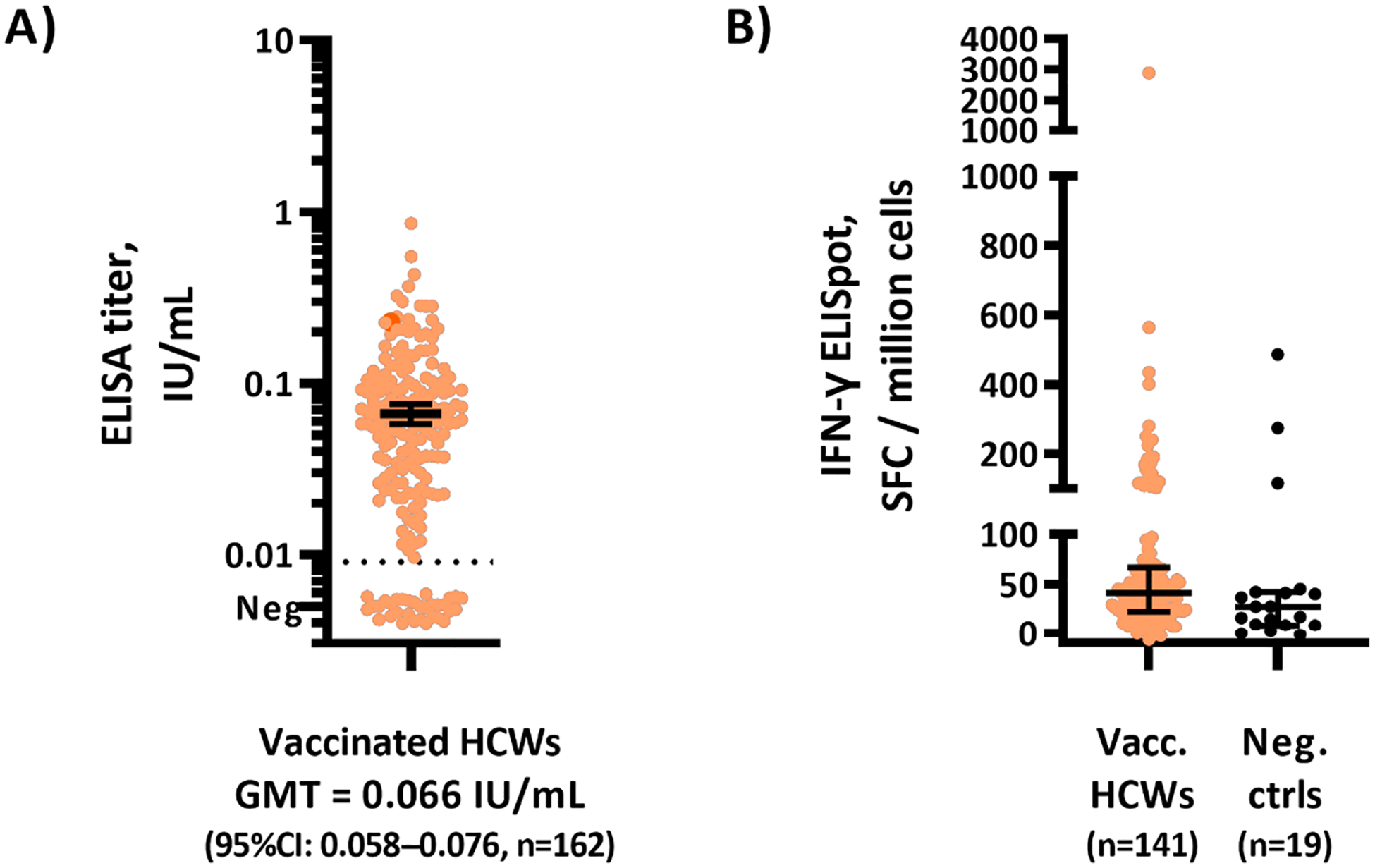
Immune responses against Ebola virus (EBOV) glycoprotein (GP) among vaccinated healthcare workers. A) IgG titers against EBOV GP trimer. Geometric mean titer and 95 % confidence intervals are presented for the seropositive healthcare workers. Negative samples (i.e., below dashed line) are nudged for illustration purposes and do not represent quantifiable titers. The single sample that neutralized EBOV (at FRNT_50_ = 58) is depicted as the red dot. B) T cell responses as measured by ELISPot detecting IFN-γ-secreting cells, with median values and interquartile ranges. No statistically significant difference between the groups was noted (Mann-Whitney test p = 0.06). All unvaccinated healthcare workers included as T cell assay negative controls tested negative for GP antibodies. Abbreviations: GMT = geometric mean titer; IU = International Unit; HCW = healthcare worker; SFC = spot-forming cell.

IgG antibodies against EBOV GP were detected in 84 % (162/194) of HCWs vaccinated with rVSV-ZEBOV. However, among the 64 vaccinated HCWs who provided a vaccination card, antibodies were detected in 95 % (n = 61) of these HCWs (z-test, p-value = 0.017). Overall, geometric mean titer among the seropositive vaccinees was 0.066 IU/mL (95 % CI: 0.058–0.076; corresponding to 1791 FANG ELISA units/mL) (Fig. 2A). Marked individual variation (IQR: 0.037–0.113, range: 0.010–0.859) resulted in the titer not being significantly correlated with time from vaccination to sample collection (Supplementary Fig. S1). Of the 352 unvaccinated HCWs, 4 % (n = 14) were seroreactive by ELISA. Rare mis-designation of a vaccinated HCW as unvaccinated appears possible considering that seropositivity was somewhat higher in HCWs with vaccination cards than among all HCWs who reported being vaccinated. All seven HCWs that reported prior exposures to confirmed or suspect Ebola disease patients but had not received the vaccine tested negative for GP antibodies.

The ability to neutralize live EBOV was detected in one vaccinated HCW sample as well as in one of the two vaccinated HCWs that had survived BDBV infection. T cell responses to EBOV GP were not statistically different in 141 vaccinated HCWs compared with 19 unvaccinated control HCWs (Fig. 2B).

## 4. Discussion

Investigation into long-term immune response following Ebola vaccination and KAP related to Ebola disease and vaccination among Ugandan HCWs is important as this population is considered high-risk due to the continued threat of re-emergence of Ebola disease in Uganda. The rVSV-ZEBOV Ebola vaccine has been shown to provide protection from Ebola disease caused by EBOV [18] and importantly, does not protect against other orthoebolaviruses [8], including SUDV and BDBV, that have caused previous Ebola disease outbreaks in Uganda [4,19]. While knowledge regarding the specifics of the rVSV-ZEBOV vaccine (e.g., that it only protects against Ebola disease caused by EBOV) was low among vaccinated and unvaccinated HCWs, most HCWs indicated that they would want to receive a booster or initial dose of rVSV-ZEBOV, respectively. IgG antibodies against EBOV GP were detected in the majority of vaccinated HCWs in Uganda 2–4 years after vaccination; however, the duration and correlates of protection warrant further investigation.

Given the species-specific protection of the rVSV-ZEBOV vaccine, it is critical that HCWs have knowledge of which orthoebolaviruses they are and are not protected against. Nearly 80 % of HCWs could not name at least one species of virus that cause Ebola disease and most HCWs did not know that rVSV-ZEBOV only protects against EBOV, although awareness of the Ebola vaccine was high. These KAP findings demonstrate the importance of continued education and risk communication to HCWs on Ebola and other viral hemorrhagic fevers (VHFs), and the need for continued vigilant infection prevention and control practices when working with patients with suspected VHFs. Further, given the proportion of these HCWs who reported occupations of nurses, midwives, doctors, or clinical, medical, or dental officers (27 %), these findings also highlight an opportunity to enhance education surrounding Ebola and other VHFs during formal clinical training in Uganda and other endemic countries. Notably, most HCWs had a positive opinion towards Ebola vaccination and indicated they would want to receive a booster or initial dose immediately. These findings are consistent with studies in other countries that have assessed HCWs’ perspective towards Ebola vaccination [20–22].

Most vaccinated HCWs had IgG antibodies against EBOV GP and higher EBOV seropositivity was observed among HCWs who provided a vaccination card. Our laboratory findings regarding the geometric mean antibody titer and seroresponse rate were in good agreement with published data from other countries where serological responses were evaluated 1–2 years after vaccination [23–26]. However, observed antibody titers vary between individuals, and the antibody titers (or other laboratory result parameters) that correlate with protection from Ebola disease have not been established despite ongoing research on the topic. WHO currently recommends that in the context of an EBOV outbreak, all contacts, and contacts of contacts of Ebola disease patients should receive a dose of rVSV-ZEBOV if they have not received vaccination in the preceding six months [27]. Given vaccine supply constraints and unknown duration of protection, widespread preventive use of rVSV-ZEBOV is not currently recommended in the absence of an outbreak. Therefore, it is recommended that all vaccinated individuals continue to observe the same stringent infection prevention measures as those who have not been vaccinated.

This investigation is subject to a few limitations. First, although we attempted to verify self-reported vaccination history, vaccination history among HCWs who could not provide a vaccination card may have been subject to recall bias. Higher seropositivity was observed among HCWs who provided a vaccination card; it is possible that vaccination status of some HCWs was miscategorized. Second, there is uncertain reliability in some reports of previous Ebola disease exposure among participants with the lack of detectable immune response that were unvaccinated. Third, single timepoint retrospective sampling limits titer stability assessment.

As Uganda has experienced outbreaks of SUDV and BDBV, for which rVSV-ZEBOV does not protect against [8], our findings underscore the importance and challenges of risk communication to HCWs, and that infection prevention and control practices must be maintained when working with patients with suspected VHFs regardless of vaccination history. While antibodies were detected in most vaccinated HCWs 2–4 years after rVSV-ZEBOV vaccination, the exact antibody titers that correlate with protection from Ebola disease remain to be determined.

## 5. Disclaimer

The findings and conclusions in this report are those of the authors and do not necessarily represent the official position of the Centers for Disease Control and Prevention.

## Supplementary Material

Appendix

## Figures and Tables

**Table 1:** Characteristics of enrolled vaccinated and unvaccinated healthcare workers in three high-risk districts in Uganda.

Characteristic	Total, n	Vaccinated, n (%)	Unvaccinated, n (%)	p-value^1^	OR (95 % CI)^2^
Health facility				<0.01	
A	67	24 (11.4)	43 (12.1)		*Reference*
B	8	8 (3.8)	0 (—)		Undefined
C	99	44 (21.0)	55 (15.5)		1.43 (0.76, 2.73)
D	127	36 (17.1)	91 (25.6)		0.71 (0.38, 1.34)
E	53	10 (4.8)	43 (12.1)		0.42 (0.17, 0.95)
F	136	62 (29.5)	74 (20.8)		1.50 (0.83, 2.77)
G	75	26 (12.4)	49 (13.8)		0.95 (0.48, 1.90)
District				<0.01	
Bundibugyo	75	32 (15.2)	43 (12.1)		*Reference*
Kabarole	180	46 (21.9)	134 (37.7)		0.46 (0.26, 0.81)
Kasese	310	132 (62.9)	178 (50.1)		1.00 (0.60, 1.67)
Median age, y (IQR)	31 (24–43)	40 (32–49)	26 (22–37)	<0.01	
Age group (years)				<0.01	
18–25	187	19 (9.0)	168 (47.3)		*Reference*
26–35	151	62 (29.5)	89 (25.1)		6.16 (3.53, 11.19)
36–45	108	58 (27.6)	50 (14.1)		10.26 (5.69, 19.22)
46–59	98	61 (29.0)	37 (10.4)		14.58 (7.94, 27.87)
≥60	21	10 (4.8)	11 (3.1)		8.04 (3.00, 21.65)
Gender				0.38	
Male	300	117 (55.7)	183 (51.5)		*Reference*
Female	265	93 (44.3)	172 (48.5)		0.85 (0.60, 1.19)
Nationality				1.00	
Ugandan	564	210 (100)	354 (99.7)		—
Tanzanian	1	0 (—)	1 (0.3)		—
Education level				<0.01	
None	4	3 (1.4)	1 (0.3)		3.49 (0.43, 71.79)
Some Primary	38	17 (8.1)	21 (5.9)		0.94 (0.44, 1.98)
Finished Primary	106	49 (23.3)	57 (16.1)		*Reference*
Finished Secondary	99	35 (16.7)	64 (18.0)		0.64 (0.36, 1.11)
Certificate Level	188	41 (19.5)	147 (41.4)		0.32 (0.19, 0.54)
Diploma, Bachelors, Masters, or Doctoral Level	130	65 (31.0)	65 (18.3)		1.16 (0.70, 1.95)
Occupation				<0.01	
Cleaner; plumber; caterer	136	60 (28.6)	76 (21.4)		*Reference*
Nurse; midwife	128	48 (22.9)	80 (22.5)		0.76 (0.46, 1.24)
Student	117	10 (4.8)	107 (30.1)		0.12 (0.05, 0.24)
Askari; security; triage; driver	38	17 (8.1)	21 (5.9)		1.03 (0.49, 2.11)
Administrator; biostatistician	30	12 (5.7)	18 (5.1)		0.84 (0.37, 1.87)
Community health worker; social worker	37	15 (7.1)	22 (6.2)		0.86 (0.41, 1.80)
Doctor; clinical, medical, or dental officer	26	14 (6.7)	12 (3.4)		1.48 (0.64, 3.48)
Laboratorian	19	14 (6.7)	5 (1.4)		3.55 (1.28, 11.48)
Theatre assistant; outpatient	8	3 (1.4)	5 (1.4)		0.76 (0.15, 3.22)
Mortuary attendant	2	1 (0.5)	1 (0.3)		1.27 (0.05, 32.47)
Pharmacist	1	1 (0.5)	0 (—)		Undefined
Other occupation^3^	23	15 (7.1)	8 (2.3)		2.38 (0.97, 6.25)
Time worked in healthcare				<0.01	
<1 year	70	2 (1.0)	68 (19.2)		0.38 (0.29, 0.50)
1–5 years	257	71 (33.8)	186 (52.4)		*Reference*
6–10 years	97	50 (23.8)	47 (13.2)		2.79 (1.72, 4.53)
>10 years	141	87 (41.4)	54 (15.2)		4.22 (2.74, 6.56)

Abbreviations: OR = odds ratio, CI = confidence interval, IQR = interquartile range.

1 Differences in frequency distribution between groups were assessed using Chi-square tests and Fischer’s exact tests when the expected cell size was < 5.

2 Undefined = undefined OR due to 0 observations in comparison group(s).

3 Other occupation includes store/inventory (n = 4), supply/cold chain (n = 3), curtain operator/attendant (n = 3), teacher (n = 2), volunteer (n = 2), tailor (n = 1), biomedical technician (n = 1), health information assistant (n = 1), messenger (n = 1), blood bank (n = 1), shop (n = 1), dark room attendant (n = 1), hospital farm caretaker (n = 1), shelter attendant (n = 1).

**Table 2:** Knowledge, attitudes, and practices related to Ebola and vaccination among healthcare workers in three high-risk districts in Uganda by Ebola vaccination status.

Characteristic	Total, n	Vaccinated, n (%)	Unvaccinated, n (%)	p-value^1^
Knowledge of one or more species of virus that cause Ebola disease				<0.01
Yes	117	70 (33.3)	47 (13.2)	
No	448	140 (66.7)	308 (86.8)	
Knowledge that Ebola vaccine only protected against Ebola virus				<0.01
Yes	76	62 (32.3)	14 (7.0)	
No	316	130 (67.7)	186 (93.0)	
Want to receive a booster (for vaccinated HCWs) or initial dose (for unvaccinated HCWs) of Ebola vaccine				0.82
Yes	518	192 (91.4)	326 (91.8)	
No or Unsure	47	18 (8.6)	29 (8.2)	
Unprotected exposure to a symptomatic, suspect, or confirmed Ebola disease patient^2^				0.29
Yes	15	8 (4.1)	7 (2.1)	
No	516	188 (95.9)	328 (97.9)	
Prior diagnosis of Ebola disease				0.14
Yes	2	2 (1.0)	0 (—)	
No	563	208 (99.0)	355 (100)	

Abbreviations: HCW = healthcare worker.

1 Differences in frequency distribution between groups were assessed using Chi-square tests and Fischer’s exact tests when the expected cell size was < 5.

2 Among the 15 HCWs who reported an unprotected exposure to blood or body fluid(s) from a symptomatic, confirmed, or suspect Ebola disease patient, five were exposed to confirmed Ebola disease patients, two were exposed to suspected Ebola disease patients that later tested negative, and eight did not know or remember the exposure patient’s final diagnosis.

## Data Availability

Data will be made available on request.
