# Supplementary material for: Knowledge, attitudes, and practices and long-term immune response after rVSVΔG-ZEBOV-GP Ebola vaccination in healthcare workers in high-risk districts in Uganda
Source: Vaccine. Author manuscript; Available in PMC 2025 Feb 20. (PMC11841019; doi:10.1016/j.vaccine.2024.05.079)
Supplement: Appendix [file NIHMS2054001-supplement-Appendix.pdf]

Data Collection Tool

CONSENT

INTRODUCTION AND PURPOSE

Uganda Virus Research Institute (UVRI), in collaboration with the U.S. Centers for Disease Control and Prevention (CDC), Emory University, and Uganda Ministry of Health, is conducting a research study among healthcare workers to evaluate risk factors for having antibodies against Ebola virus and other hemorrhagic fever viruses and investigate immune responses following vaccination in those that received the rVSVΔG-ZEBOV-GP Ebola vaccine (referred to as Ebola vaccine moving forward). To do this, we are recruiting individuals working as healthcare workers in participating facilities in high-risk districts in Uganda, including those who have been vaccinated with the Ebola vaccine.

As an eligible individual, your participation in this study would be very helpful for our research. For this reason, we are inviting you to participate in this research.

PROCEDURES

We will collect a maximum of 42ml of blood (about 3 tablespoons) from you through a vein in your arm to assess the immune response after vaccination with the Ebola vaccine. If you indicate that you have received the Ebola vaccine, we may request to revisit and collect the same amount of blood at additional different time points approximately 30, 36, 42, 48, 54, and 60 months after vaccination. In addition, we will collect basic information about you, specifically: age, gender, occupation, when you were vaccinated with the vaccine; whether you recall having any exposures to Ebola virus after you were vaccinated; and whether you have received any other experimental vaccines against Ebola virus. This information will be used to interpret your lab results. At each specimen collection visit we will ask whether you recall having any exposures to Ebola since your last visit, and whether you have received any other vaccines against Ebola. We may learn things that might be related to your health as part of the research, however we will not share this information with you because we do not know how true this information is. We are also learning about the tests used, and we do not want to provide wrong information.

It is also possible that your information might be de-identified and used for future research at UVRI or CDC or shared with outside researchers without your knowledge.

RISKS

The study presents minimal risk to you. You may experience pain for a short time during blood sample collection. There are very minimal risks associated with having your blood drawn, including excessive bleeding, fainting, or feeling lightheaded, hematoma, and infection.

BENEFITS

The information we collect in our study will not help you directly but will help us better understand immune responses following vaccination.

CONFIDENTIALITY

To maintain your privacy, we will keep your study records under a study number. Your name will be stored separately. Links between your name and study ID will be kept in a secure, password-protected database. Only study staff will have access to this information. Maintaining a link between your name and study ID will allow us to identify and destroy your specimens in the event you wish to withdraw from the study. We will keep the records, including paper records and digital files, in locked files. Only staff from this study will be allowed to look at them. The link between your name and the study ID will be destroyed once the specimens are tested. Your name or other facts that might point to you will not appear when we report the findings of this study.

COST/PAYMENT

Participating in this study is completely voluntary. If you agree to participate, you will receive 10,000 UGX to compensate

you for your time to participate in this study. If you do not participate, it will not affect any your relationship with your supervisor(s), your employer, the Ministry of Health, or any of the other participating institutions conducting this study.

RIGHT TO REFUSE OR WITHDRAW

You are free to participate in this study or not participate. You can withdraw your consent for participation in this study at any time. You may also decline to participate or withdraw without penalty. If you decide to withdraw from the study after you have been enrolled, your remaining samples will be destroyed but we will keep any information that has already been collected or generated.

PERSONS TO CONTACT

If at any time you have questions about the study or feel you may have been harmed by participating in the study, you may contact Dr. Luke Nyakarahuka, one of study's primary investigators.

Dr. Luke Nyakarahuka
Email: XXXX@gmail.com
Phone number: XXXX XXXXXX

If you have questions about your rights as a research subject or if you feel that you have been pressured to participate in this study, you may contact the Uganda Virus Research Institute Research and Ethics Committee Chairman at Telephone Number XXXXXXXXXX.

I agree to answer the survey and give a blood sample ☐ Yes ☐ No
I agree to the storage of samples for future research ☐ Yes ☐ No
I agree to be contacted regarding future follow-up visits for this study ☐ Yes ☐ No
If yes, phone number: ______________________

Participant Electronic Signature: ________________________________
Participant first name: ______________________
Participant middle name: ______________________
Participant surname: ______________________

INTERVIEWER INFORMATION

Interview/Specimen Collection Date: _________________________
Interviewer name (drop-down):

☐ Luke ☐ Jimmy ☐ Sophia ☐ Stephen ☐ Alex ☐ Jackson
☐ Michelle ☐ Caitlin ☐ Amy ☐ Markus ☐ Other

If 'Other', interviewer name: ________________________
If 'Other', interviewer phone number: _____________________
If 'Other', interviewer e-mail: ________________________

Interviewer Signature: _________________________________

SECTION I: DEMOGRAPHIC DATA OF PARTICIPANT

1. Participant ID: _________________________
   ☐ Please check this box if the participant ID needs to be edited
2. Age: _____ years
3. Gender: ☐ Male ☐ Female
4. Nationality:
   ☐ Ugandan ☐ Congolese ☐ Rwandan ☐ South-Sudanese
   ☐ Kenya ☐ Tanzanian ☐ Indian ☐ Other, specify: _________________
5. Current health facility (drop-down):

☐ Kagando Hospital ☐ Bwera Hospital ☐ Kilembe Hospital ☐ Ft Portal Reg Ref Hospital
☐ Kabarole Hospital ☐ Bundibugyo Hospital ☐ Busaru HCIV ☐ Nyahuka HCIV
☐ Other, specify: _______________

6. How long have you worked at this health facility?
   ☐ <1 year ☐ 1-5 years ☐ 6-10 years ☐ >10 years
7. How long have you worked as a healthcare worker?
   ☐ <1 year ☐ 1-5 years ☐ 6-10 years ☐ >10 years
8. What is your current position/designation within the facility?
   ☐ Clinical officer ☐ Enrolled nurse ☐ Midwife ☐ Administrator
   ☐ Records assistant ☐ Biostatistician ☐ Secretary ☐ Askari/security
   ☐ Nurse ☐ Medical superintendent ☐ Doctor ☐ Medical officer
   ☐ Accountant ☐ Laboratory technician ☐ Cleaner ☐ Laboratory assistant
   ☐ Pharmacist ☐ Mortuary attendant ☐ Student ☐ Driver
   ☐ Other, specify: _________________
9. Education Level:
   ☐ Primary (P7) ☐ Ordinary (S4) ☐ Advanced Level (S6) ☐ Certificate Level
   ☐ Diploma ☐ Bachelors Level ☐ Masters Level ☐ Doctoral Level
   ☐ Other, specify: ______________

SECTION II: PARTICIPANT VACCINATION HISTORY

10. Have you received Ebola vaccination? ☐ Yes ☐ No ☐ Unsure

    If yes or unsure to Question 10:

    a. Do you have a vaccination card with you? ☐ Yes ☐ No

       If yes to Question 10a:

       May we please see the vaccination card? ☐ Yes ☐ No

    If yes to Question 10:

    b. Which kind of Ebola vaccine did you receive?
       ☐ rVSVΔG-ZEBOV-GP Ebola vaccine (Merck vaccine, one dose)
       ☐ Johnson and Johnson [Janssen] (two doses)
       ☐ Chimpanzee adenovirus vector vaccine (two doses)
       ☐ Other (specify): ______________
       ☐ Unknown
    c. When did you receive the Ebola vaccine?
       i. Year
       ii. Month
       iii. Day
    d. Were you working in this health facility when you received the Ebola vaccine?
       ☐ Yes ☐ No ☐ Unsure

       If no to Question 10d:

       i. Which other health facility were you working in when you received the Ebola vaccine? ________

If no or unsure to Question 10:

11. Were you ever offered the Ebola vaccine?
    ☐ Yes, but I did not take it ☐ No, I was not offered ☐ Unsure

If yes to Question 11:

12. Why did you not take the Ebola vaccine?
    ☐ I did not have time ☐ Family or peer influence
    ☐ Safety (I fear the side effects, impact on family planning) ☐ Efficacy (I do not think they work)
    ☐ Risk perception (I am not at risk of Ebola or I do not believe Ebola is real)

☐ Mistrust (of government, foreign manufacturers) ☐ My faith or culture does not allow vaccination
☐ Contraindications (I have allergies or history of vaccine reactions) ☐ Other, specify: ________________

SECTION III: RISK FACTORS FOR SEROPOSITIVITY

13. When you are working in the health facility, do you typically provide patient care or handle materials used by patients? ☐ Yes ☐ No ☐ Unknown

If yes to Question 13:

a. What duties do you typically perform? (check all that apply)
☐ Direct patient care ☐ Sample handling (collection, processing, testing)
☐ Cleaning of patient linens ☐ Waste handling/management/cleaning of facility
☐ Pharmacy ☐ Patient transport ☐ Triage / security
☐ Mortuary attendant ☐ Other, specify: _________________________

b. Do you normally use personal protective equipment (PPE) when performing these activities?
☐ Yes ☐ No ☐ Unknown

If yes to Question 13b:

i. What kind of PPE do you typically wear when you are performing these activities? (check all that apply)
☐ Gloves ☐ Cloth or surgical style mask ☐ Respirator (e.g. N95)
☐ Face shield/goggles ☐ Gum boots or boot covers ☐ Disposable apron or gown/suit
☐ White coat ☐ Other, specify: ______________________________

ii. How often do you use PPE when performing these activities?
☐ Rarely ☐ Sometimes ☐ Always ☐ Other, specify: _____________

If no to Question 13b:

i. Why do you not typically wear PPE? (check all that apply)
☐ It is not available (my facility has challenges sourcing PPE) ☐ I do not have time to use PPE
☐ I am not trained to properly use PPE to protect against infectious diseases
☐ I do not need PPE to protect myself against infectious diseases ☐ Other, specify:_____________

14. Have you ever had unprotected exposure(s) to blood or body fluids (e.g., vomit, stool, semen, urine, saliva, post-partum fluids/tissues, etc) from a patient suspected to have **an infectious disease**?
☐ Yes ☐ No ☐ Unknown

If yes to Question 14:

a. What type of exposure have you had to blood or body fluids of ill patients? (Check all that apply)
☐ Contact of blood or body fluids with your intact skin
☐ Contact of blood or body fluids with your broken skin (fresh cut, burn, abrasions)
☐ Contact of blood or body fluids with your mucous membranes (eyes, nose, mouth, genitals)
☐ Contact of blood or body fluids with via a needle stick
☐ Other, specify____________________________________

b. When was your last exposure?
i. Year
ii. Month

15. Have you ever had unprotected exposure(s) to blood or body fluid(s) from a **symptomatic, confirmed or suspect EVD patient**? ☐ Yes ☐ No ☐ Unknown

If yes to Question 15:

a. When was the exposure to the suspect or confirmed EVD patient?
i. Year
ii. Month

b. What type of exposure did you have to the suspect or **confirmed EVD patient?** (Check all that apply)
☐ Contact of blood or body fluids with your intact skin

☐ Contact of blood or body fluids with your broken skin (fresh cut, burn, abrasion)
☐ Contact of blood or body fluids with your mucous membranes (eyes, nose, mouth, genitals)
☐ Contact of blood or body fluids with via a needle stick
☐ Other: Specify___________________________________

c. What body fluid(s) of the suspect or confirmed EVD patient were you exposed to? (Check all that apply)
☐ Blood ☐ Vomit ☐ Stool ☐ Saliva ☐ Sweat ☐ Urine ☐ Tears
☐ Breast milk ☐ Semen ☐ Vaginal fluid ☐ Post-partum fluids/tissues
☐ Respiratory/Nasal secretions ☐ Cerebral spinal fluid ☐ Other, specify: ________________

d. Explain how the exposure happened (free text):

16. Have you ever been diagnosed with EVD? ☐ Yes ☐ No ☐ Unknown

If yes to Question 16:

a. When were you diagnosed (year)?
b. Could you please provide details about when you were diagnosed with EVD? (e.g., how and where you were infected, if you were hospitalized, what your symptoms were, etc.) [free text]

SECTION IV: EVD and VACCINE KNOWLEDGE, ATTITUDES, AND PRACTICES

17. Which vaccines do you routinely take as an adult (>18 years of age)?

a. COVID-19? ☐ Yes ☐ No ☐ Unknown
b. Tetanus? ☐ Yes ☐ No ☐ Unknown
c. Yellow Fever? ☐ Yes ☐ No ☐ Unknown
d. Rabies? ☐ Yes ☐ No ☐ Unknown
e. Influenza? ☐ Yes ☐ No ☐ Unknown
f. Hepatitis B? ☐ Yes ☐ No ☐ Unknown
g. Other? ☐ Yes ☐ No ☐ Unknown

If Other, specify: __________

If yes or unsure to Question 17a (COVID-19 vaccine):

i. Do you have a vaccination card with you? ☐ Yes ☐ No

If yes:

May we please see the vaccination card? ☐ Yes ☐ No

If yes to Question 17a (COVID-19 vaccine):

ii. Which COVID-19 vaccine did you receive?
☐ Astrazeneca ☐ Moderna ☐ Pfizer ☐ Johnson and Johnson
☐ CoronaVac ☐ Sinovac ☐ Sinopharm ☐ Unknown
☐ Other, specify: _________________

iii. How many doses of the COVID-19 vaccine have you received?
☐ One ☐ Two ☐ Unknown

iv. When did you receive your last COVID-19 vaccine?
1. Year
2. Month
3. Day

If no to Question 17a (COVID-19 vaccine), why did you not take the COVID-19 vaccine? (check all that apply)
☐ I don't have time ☐ I would like to, but vaccines aren't available ☐ Family or peer influence
☐ Safety (I fear the side effects, impact on family planning) ☐ Efficacy (I do not think they work)
☐ Risk perception (I do not think they are necessary to be healthy)
☐ Mistrust (of government, foreign manufacturers) ☐ My faith or culture does not allow vaccination
☐ Contraindications (I have allergies or history of vaccine reactions) ☐ Other, specify: ____________

18. Do you know the different species of viruses that cause Ebola virus disease? ☐ Yes ☐ No

If yes to Question 18:

a. Please name the different species of viruses that cause Ebola virus disease:

Note to interviewer: do not read off answer choices. Please check all that participant can name.

☐ *Zaire ebolavirus* ☐ *Sudan ebolavirus* ☐ *Bundibugyo ebolavirus*

☐ *Tai forest ebolavirus* [formerly *Cote d'Ivoire ebolavirus*] ☐ *Reston ebolavirus*

☐ *Bombali ebolavirus*

19. Did you know before this survey that there was a vaccine to protect against Ebola? ☐ Yes ☐ No

If yes to Question 19:

a. Do you know which species the Ebola vaccine protects against?

Note to interviewer: do not read off answer choices. Please select the answer choice based on the participant's response.

☐ All of them ☐ None of them ☐ Only *Zaire ebolavirus*

☐ Unknown ☐ Other, specify: _________________

20. Did you know that healthcare/frontline workers in Uganda were vaccinated against Ebola in 2018-2019?

☐ Yes ☐ No

21. ***For unvaccinated individuals only***: if you were given the opportunity in the future, would you take the Ebola vaccine? ☐ Yes ☐ No ☐ Unsure

If no or unsure to Question 21:

a. Why don't you want to be vaccinated against Ebola? (Select all that apply)

☐ I don't have time ☐ I would like to, but vaccines aren't available ☐ Family or peer influence

☐ Safety (I fear the side effects, impact on family planning) ☐ Efficacy (I do not think they work)

☐ Risk perception (I am not at risk of Ebola or I do not believe Ebola is real)

☐ Mistrust (of government, foreign manufacturers) ☐ My faith or culture does not allow vaccination

☐ Contraindications (I have allergies or history of vaccine reactions) ☐ Other, specify: ________________

If yes to Question 21:

b. When would you ideally receive the vaccine? Check all that apply.

☐ Immediately ☐ After an Ebola case is identified in DRC

☐ After an Ebola case is identified somewhere in Uganda ☐ After an Ebola case is identified in my district

☐ After an Ebola case is identified in my healthcare facility

☐ After I receive more education about the vaccine (safety/efficacy) ☐ Other, specify: ___________________

c. Why do you want to be vaccinated against Ebola? Check all that apply.

☐ Protection/immunity (protection from Ebola, protection from signs/symptoms, protection from illness, protection from virus

☐ I understand more about the vaccine now (more education received about the vaccine)

☐ It wasn't available, but I want it now (prior contradiction, no vaccine available, missed initial offering of vaccine)

☐ It is required for my job

☐ Other (specify):

22. ***For vaccinated individuals only***: if you were given the opportunity in the future, would you take a booster vaccine against Ebola? ☐ Yes ☐ No ☐ Unsure

If no or unsure to Question 22:

a. Why don't you want to receive a booster vaccine against Ebola? (Select all that apply)

☐ I don't have time ☐ I would like to, but vaccines aren't available ☐ Family or peer influence

☐ Safety (I fear the side effects, impact on family planning) ☐ Efficacy (I do not think they work)

☐ Risk perception (I am not at risk of Ebola or I do not believe Ebola is real)

☐ Mistrust (of government, foreign manufacturers) ☐ My faith or culture does not allow vaccination

☐ Contraindications (I have allergies or history of vaccine reactions) ☐ Other, specify: ________________

If yes to Question 22:

a. When would you ideally receive the booster vaccine? Check all that apply.

□ Immediately □ After an Ebola case is identified in DRC
□ After an Ebola case is identified somewhere in Uganda □ After an Ebola case is identified in my district
□ After an Ebola case is identified in my healthcare facility
□ After I receive more education about the vaccine (safety/efficacy) □ Other, specify: ___________________

b. Why do you want to receive a booster vaccine against Ebola (free text)? Check all that apply.
c. □ Protection/immunity (protection from Ebola, protection from signs/symptoms, protection from illness, protection from virus
d. □ I understand more about the vaccine now (more education received about the vaccine)
e. □ It wasn't available, but I want it now (prior contradiction, no vaccine available, missed initial offering of vaccine)
f. □ It is required for my job
g. □ Other (specify):
